# Silver Thickness Optimization for Sensitivity Enhancement in a Surface Plasmon Resonance Biosensor for Multi-Cancer Detection

**DOI:** 10.3390/s26216954

**Published:** 2026-11-03

**Authors:** Rajeev Kumar, Biswajit Brahma, Jitendra Singh Tamang, Paolo Barsocchi, Akash Kumar Bhoi

**Affiliations:** 1 Department of Electronics and Communication Engineering, Graphic Era (Deemed to Be University), Dehradun 248001, Uttarakhand, India; 2 McKesson Corporation, 32559 Lake Bridgeport St, Fremont, CA 94555, USA; 3 Department of Electronics and Communication Engineering, Sikkim Manipal Institute of Technology, Sikkim Manipal University, Majitar 737136, Sikkim, India; jitendra.t@smit.smu.edu.in; 4 Institute of Information Science and Technologies, National Research Council, 56124 Pisa, Italy; 5 Symbiosis Institute of Technology, Pune Campus, Symbiosis International (Deemed University), Pune 412115, India

**Keywords:** surface plasmon resonance, cancer, sensitivity, detection accuracy, electric field

## Abstract

This work proposes a highly sensitive Surface Plasmon Resonance (SPR) biosensor for label-free refractive index (RI) and biological sensing applications based on a BK7 prism-coupled bimetal (Ag/Au) and Tantalum Diselenide (TaSe_2_) hybrid structure. The transfer matrix method (TMM) is used to examine the sensing performance of the suggested structure while accounting for the effects of various biological sensing media (SM) and plasmonic layer thickness. To attain optimal plasmonic coupling, the impact of Ag layer thickness on sensitivity and minimum reflectance (R_min_) is methodically examined. For the long RI range of 1.35–1.40, the maximum sensitivity is 206.32°/RIU at the R_min_ value. Depending on the sensing layer, the Ag thickness ranges from 39 to 42 nm, offering better resonance properties for various cancer detection. Basal, HeLa, Jurkat, PC12, MM-231 and MCF-7 have remarkable Rmin values and the highest sensitivity of 260.49°/RIU, 280.13°/RIU, 290.24°/RIU 260.49°/RIU, 280.13°/RIU and 290.24°/RIU with an optimized Ag thickness of 42–39 nm among the biological layers under investigation, respectively. Moreover, the penetration depth (PD) of 192.94 nm and 181.5 nm is attained at RIs of SM 1.35 and 1.40. The suggested SPR biosensor is a promising platform for biosensing, disease diagnosis, and real-time monitoring applications because of its exceptional sensitivity, low resonance reflectance, and improved detection capability.

## 1. Introduction

Cancer has become one of the world’s leading causes of death in recent years. According to estimates from the World Health Organization (WHO), cancer kills about 10 million people worldwide [1,2]. Over 8.3 billion people are expected to live on Earth by 2025, and over 20 million new cases of cancer are expected to occur each year [3,4,5]. The unchecked and rapid growth of abnormal cells is the hallmark of the disease known as cancer. These cells swiftly spread throughout the body, damaging numerous organs, including important organs and healthy tissue, and eventually resulting in death. Jurkat (blood cancer), HeLa (cervical cancer), PC12 (adrenal gland cancer), MDA-MB-231 (breast cancer type 1), MCF-7 (breast cancer type 2), and basal (skin cancer) are the most common types of cancer [6,7]. The outermost layer of the epidermis develops basal cancer (skin) cells due to prolonged sun exposure. To detect cancer early and reduce mortality, researchers have developed biosensor screening tests, and biosensing applications have advanced to differentiate cancer cells from healthy cells. The multi-step process of cancer involves genetic and epigenetic changes that upset the balance between cellular growth and death. Every year, cancer claims the lives of millions of people worldwide. The International Agency for Research on Cancer estimates that there are roughly 18.1 million cancer cases and 9.6 million cancer-related deaths annually [8,9]. One of the most significant worldwide health issues that contributes to morbidity and mortality in developed nations is cancer. Molecular and tissue-level alterations that promote malignant abnormalities throughout the tissue may be linked to significant factors in the development of cancer. Examining the biomolecules involved in the molecular pathogenesis of cancer may provide valuable clinical insights, i.e., biomarkers, which are essential for determining whether cancer is suspected [10,11]. These molecules include proteins, lipids, carbohydrates, nucleic acids, and metabolites. Biomarkers can serve many purposes, such as assessing a person’s risk of developing cancer, predicting the likelihood that a particular drug will work for a given patient, and monitoring the course of treatment to determine its efficacy. To reduce cancer mortality and morbidity, reliable and validated cancer markers are desperately needed. In addition to identifying cancer, biomarkers also classify it by type and stage. SPR is a powerful analytical technique for real-time, label-free detection of biomolecular interactions. This technique uses light interaction with a thin metal sheet to measure changes in RI at the sensor surface [12,13]. By immobilizing one biomolecule on the sensor chip and then adding a second biomolecule, researchers can precisely measure binding kinetics and affinity. Compared with traditional methods, this technology offers numerous advantages, including high sensitivity, real-time interaction monitoring, and the elimination of labeling processes. Biosensing, diagnostics, and drug development are just a few of the fields in which SPR has found extensive application [14,15,16]. A SPR sensor is considered to be in resonance when the wave vector of the incident light and the surface plasmon wave (SPW) moving along the metal–dielectric interface coincide [17,18,19,20,21]. This phase-matching requirement can be satisfied by changing the angle of incidence or the RI of the dielectric medium. When resonance is reached, SPs are excited, as shown by a sharp drop in reflected light intensity. Otto and Kretschmann invented the field of SPR to generate SPWs [22,23]. In the early 1970s, they independently developed the theoretical underpinnings and experimental techniques for SP stimulation and detection. Otto used a prism to couple light into the metal layer [23], while Kretschmann used a thin metal layer deposited on a prism [22]. Kretschmann arrangement has many advantages over Otto’s. Direct contact between the prism and the metal layer allows more efficient light coupling, resulting in higher sensitivity. Because it is usually easier to set up in a lab and more portable, the Kretschmann structure is a popular choice in SPR research. SPR sensors require gold (Au), silver (Ag), Copper (Cu), and other plasmonic metals to excite SP [24,25,26]. Because of their unique optical properties, these metals can maintain collective electron oscillations at the metal–dielectric interface. Several factors influence the choice of plasmonic metal, including the desired wavelength range, chemical stability, and biocompatibility [27]. Au is often used in biological applications because of its biocompatibility and durability, but Ag may be better suited for applications requiring high sensitivity in the visible spectrum [28]. Ag has several advantages over Au for SPR applications [19]. It has a better plasmonic response, which boosts sensitivity, and supports SPs across a wider spectral range. Moreover, Ag is usually less costly than Au. However, because it is more susceptible to oxidation, it requires proper surface protection. SPR biosensors offer advantages, but practical application in cancer-related sensing remains limited by several challenges. Conventional single-metal SPR structures typically involve trade-offs among plasmonic coupling strength, optical damping, resonance linewidth, and chemical stability. Ag’s susceptibility to oxidation and surface degradation can affect long-term performance, despite its high sensitivity and strong plasmonic excitation. Au may exhibit different damping and coupling properties, but it offers improved chemical stability. Practical performance also depends on FWHM, DA, surface contamination, non-specific adsorption, and reproducibility, so high sensitivity alone does not ensure accurate sensing. Target-specific cancer identification also requires suitable surface functionalization and biorecognition elements; RI-based SPR responses do not inherently provide molecular selectivity. These limitations motivate the investigation of hybrid metallic and 2D-material architectures that can simultaneously improve analyte interaction, field confinement, stability, and plasmonic coupling. An SPR-based biosensor consists of an SPR sensor and suitable surface functionalization acting as the biorecognition component. When a biomolecular interaction occurs, such as specific analyte binding, the RI near the surface changes. The SPR sensor then detects this RI shift. Both the intrinsic optical performance of the SPR sensor and the properties of the surface functionalization influence the biosensor’s overall performance because an SPR sensor and appropriate surface functionalization are essential parts of an SPR biosensor [17,18,19]. To give biosensors specific capabilities and enhance their performance, the biosensing interface must be functionalized. Integrating two-dimensional (2D) materials with conventional SPR setups offers several advantages, including increased sensitivity, tunable plasmon resonances, a broader spectral range, flexibility and scalability, and integration with other features. These materials can significantly improve SPR sensor capabilities and performance, opening new applications across a variety of industries and medical diagnostics. Because of their distinctive electronic, optical, and surface properties, 2D materials have attracted significant interest recently. This makes them attractive options for cutting-edge SPR sensing platforms. These atomically thin materials improve interactions between the sensing layer and target analytes because they have a large specific surface area, many active sites, high carrier mobility, and strong light-matter interaction. To improve SPR sensor performance, researchers have extensively investigated materials such as graphene, black phosphorus, MXenes, transition metal dichalcogenides (TMDs), and other layered nanomaterials [29,30,31,32]. Combining 2D materials with conventional plasmonic metals like Ag and Au can improve resonance sensitivity, charge transfer, and electromagnetic field confinement. TaSe_2_ 2D materials have become a popular sensing material among TMDs because of their strong molecular adsorption capacity, tunable optical properties, and high electrical conductivity. At the metal–analyte interface, the ultrathin TaSe_2_ layer can successfully alter the local dielectric environment, causing a notable shift in SPR resonance [33,34]. Furthermore, it is well suited for biomolecular recognition and RI-based detection because of its chemical stability and surface-functionalization capacity. As a result, integrating 2D materials—particularly TaSe_2_—into SPR structures offers a practical way to create highly sensitive, precise, and reliable sensors for biomedical, environmental, and biochemical applications. The sensing performance of a bimetallic SPR layer depends on the balance between optical damping and plasmonic excitation. Ag generally provides stronger plasmon excitation and higher sensitivity, while Au is more chemically stable and resistant to oxidation. However, increasing the thickness of either metal can also increase optical damping and broaden the resonance linewidth. Therefore, it is not possible to select Ag/Au thicknesses based solely on maximum sensitivity. Additionally, the resulting R_min_, FWHM, DA, and FoM must be considered. The Au layer in the proposed structure serves as a protective overlayer while also altering the electromagnetic coupling between the Ag layer and the SM.

This work proposes a bimetallic SPR sensor based on a BK7 prism for highly sensitive RI and biological sensing applications. To get around the drawbacks of conventional SPR sensors, the combination of a 2D TaSe_2_ layer and a bimetallic Ag/Au layer is investigated. While the Au layer enhances structural stability and prevents Ag oxidation, the Ag layer provides strong plasmon excitation, higher sensitivity, and greater electromagnetic-field intensity. TaSe_2_’s large surface area, superior optical qualities, and high adsorption capability further improve analyte interaction when integrated as an active sensing layer. By optimizing the thickness of the Ag and Au layers, the TMM is used to theoretically investigate the sensing performance of the suggested configuration. Sensitivity and other performance metrics are systematically examined in relation to various sensing-layer RIs. The suggested sensor exhibits enhanced resonance response, improved plasmonic coupling, and high sensitivity, suggesting its potential for label-free RI sensing and cancer cell detection. Graphene, MoS_2_, WS_2_, and other transition-metal dichalcogenides have garnered significant attention among the 2D materials examined for SPR sensing because of their strong light-matter interaction and surface activity. However, the electronic properties and optical response of each material are strongly influenced by thickness, wavelength, and the surrounding dielectric environment. In this study, we choose TaSe_2_ for its layered structure, high carrier mobility, strong light–matter interaction, and active surface sites. These features can enhance electromagnetic-field localization and analyte interaction at the sensing interface. Unlike a passive 2D-material coating, the ultrathin TaSe_2_ layer in the proposed architecture serves as an active sensing interface that alters the local dielectric environment. Thus, the objective is to leverage the complementary plasmonic response of Ag/Au and the surface and optical properties of TaSe_2_.

Two complementary aspects of biosensor operation are cancer-specific biomarker recognition and RI-based SPR sensing. While molecular specificity is typically introduced through suitable surface functionalization, the intrinsic SPR structure primarily converts changes in the local RI at the sensing interface into measurable resonance-angle or reflectance shifts. The sensing surface can be functionalized with appropriate biorecognition elements, such as receptors, aptamers, antibodies, or other selective recognition molecules, to enable target biomarker interaction in cancer-related applications. These biomolecular interactions can produce a detectable SPR response by altering the local dielectric environment near the plasmonic interface. Molecular recognition kinetics and explicit biomarker binding chemistry are not modeled in the current theoretical study. Instead, variations in the SM’s effective RI represent the biological sensing response. As a result, the current study mainly assesses the RI-sensing capacity, electromagnetic-field confinement, and plasmonic performance of the proposed Ag/Au/TaSe_2_ structure; future work should focus on target-specific cancer biomarker identification and experimental biochemical validation. Although numerous reports describe SPR sensors using single metals, bimetallic layers, and 2D materials, few designs simultaneously prioritize sensitivity enhancement. However, these designs do not simultaneously examine the impact of metal-layer optimization, resonance sharpness, electromagnetic-field confinement, and fabrication tolerance. This work investigates a hybrid Ag/Au/TaSe_2_ architecture in which ultrathin TaSe_2_ serves as the active sensing interface, Au protects against Ag degradation, and Ag enables efficient plasmon excitation. The proposed structure is assessed not only by sensitivity but also by evaluating R_min_, FWHM, detection accuracy, FoM, PD, and constituent-layer thickness tolerance. This combined analysis provides a more comprehensive assessment of the proposed hybrid configuration’s theoretical sensing performance.

## 2. Structure, Refractive Index, Mathematical Modeling and Performance Matrices

### 2.1. Proposed Structure and Role of Layers

**Figure 1:**
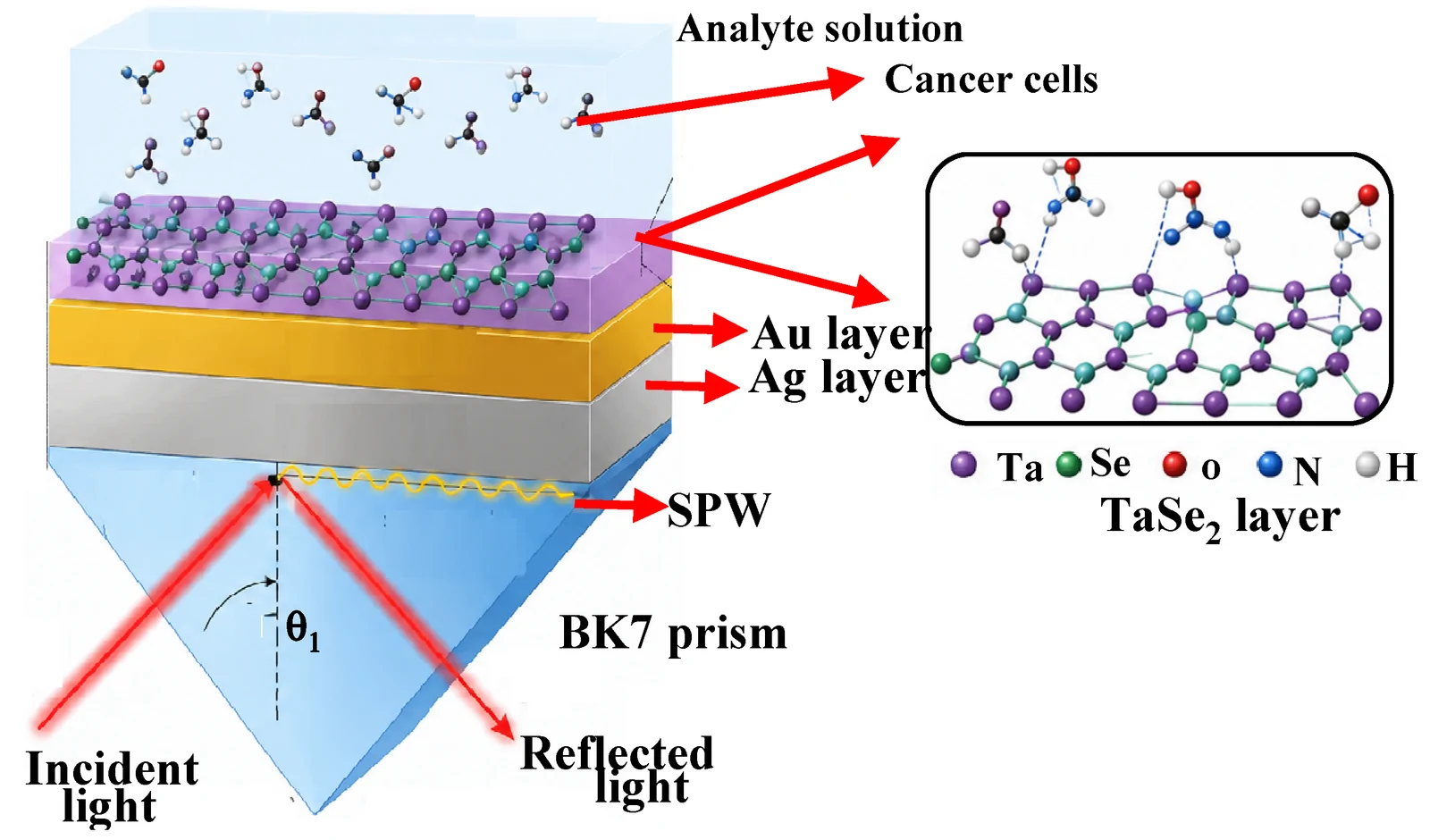
Proposed structure for the detection of various cancers.

Figure 1 shows the proposed SPR sensor configuration using the Kretschmann excitation technique for detecting various cancers. The multilayer structure comprises an Ag plasmonic layer, a thin Au protective layer, and a TaSe_2_ layer that serves as the active sensing interface exposed to the cancer analyte solution. The BK7 prism serves as the optical coupling medium. Under resonance, incident p-polarized light traveling through the BK7 prism stimulates surface plasmons at the Ag/Au interface, generating a strong electromagnetic field near the sensing surface. The Au layer enhances chemical stability and prevents Ag oxidation, while the Ag layer efficiently excites plasmons. Because of its high surface activity, the TaSe_2_ layer increases analyte adsorption and gives cancer cells more places to interact. The SPR resonance angle shifts as cancer cells interact with the TaSe_2_ surface and change the RI near the metal–dielectric interface. The reflectance curve tracks the resonance variation; as the number of cancer cells increases, the resonance minimum shifts proportionally. As a promising platform for sensitive cancer cell detection, the hybrid Ag/Au/TaSe_2_ structure offers improved sensitivity, enhanced field confinement, better molecular interaction, and a high FoM.

### 2.2. Refractive Index of Constituent Layers

The RI of each layer significantly influences SPR excitation conditions and sensing performance. RI refers to a material’s ability to interact with incident electromagnetic waves and slow their propagation. When the incident p-polarized light’s wave vector and the SP wave vector at the metal–dielectric interface coincide, an SPR sensor reaches resonance. Any change in the analyte medium’s RI modifies the momentum-matching condition, shifting the SPR resonance angle. With a RI of 1.5151 at 633 nm [27], BK7 is used as the coupling prism in the proposed structure, providing effective light coupling for plasmon excitation. Due to their metallic nature and optical losses, the dielectric functions of the Ag and Au layers are typically described using complex RIs [27]. Strong light-matter interaction gives the 1 nm-thick TaSe_2_ layer a high complex RI, enhancing electromagnetic field confinement at the sensing interface. Table 1 shows the RI and thickness of the materials used in the proposed structure. Biological cell layers with varying RI values that correspond to healthy and cancerous cells make up the SM. Their effective RIs differ because cellular composition varies, including intracellular structure, membrane properties, and protein concentration. Because of increased cellular density, altered metabolism, and increased biomolecular content, cancerous cells typically have higher RIs than healthy cells. The analyte RIs, which represent various biological cell types, are expected to range from roughly 1.360 to 1.401 in the proposed SPR sensor. Based on their cellular properties, Table 1 lists representative cell lines such as Basal, HeLa, Jurkat, PC12, MCF-7, and MM-231 with their corresponding RIs. These biological layers interact with the TaSe_2_ sensing interface, shifting the SPR resonance angle and changing the effective RI near the metal–dielectric boundary. Because cancerous cells interact more strongly with the evanescent electromagnetic field, a higher RI is linked to a greater resonance shift. To optimize important sensing parameters, such as Sensitivity, Detection Accuracy (DA), Full Width at Half Maximum (FWHM), and FoM, as well as to achieve reliable discrimination between healthy and cancerous cell types, precise estimation of the RI values of various biological cells is crucial.

For the theoretical SPR analysis, the RI values utilized for the biological sensing media in this investigation are regarded as effective RI parameters. These values are used to assess the corresponding shift in the SPR resonance angle and to represent various dielectric conditions at the sensing interface. Because the composition and dielectric characteristics of biological cells and cellular media determine their optical response, changes in effective RI alter the momentum-matching condition between the incident optical wave and the surface plasmon wave, causing a detectable shift in the resonance response. The corresponding concentration levels regarded as simulation conditions for the biological SM are indicated by the percentage values listed in Table 2. These percentages are not meant to reflect experimentally measured biomarker concentrations; rather, they differentiate the sensing conditions under investigation. As a result, rather than experimentally verified concentration–RI calibration data, the RI and percentage values in Table 1 serve as theoretical input parameters for assessing the proposed SPR sensor’s RI sensitivity. This investigation consistently identifies basal, HeLa, Jurkat, PC12, MM-231, and MCF-7 as the biological sensing media. These SM incorporate the theoretical analysis to examine how the proposed SPR sensor responds to various effective RI conditions. Rather than being experimentally measured quantities obtained in the current work, the corresponding RI values are used as simulation inputs. They represent the optical parameters of the sensing media. Therefore, it is not appropriate to interpret the terms used to describe these biological media as direct proof of cancer-specific identification; rather, they indicate the sensing conditions considered in the optical model. This clarification is crucial because, while biological classification and selective cancer recognition necessitate theoretical validation and suitable biochemical recognition mechanisms, the current study assesses RI-dependent plasmonic sensing performance.

### 2.3. Mathematical Modeling Used in SPR Sensor

The TMM under the Kretschmann configuration is used to analyze the optical response of the proposed structure. Incident p-polarized light enters through the BK7 prism and excites surface plasmons at the metal–dielectric interface. The dielectric properties and layer thicknesses determine the resonance condition.

The reflectivity (R) was determined using the TMM, which relates the electric and magnetic fields at each interface. The Fresnel equations and TMM were used to calculate the reflectance curve and sensitivity for a small change in the RI of an analyte. The reflectivity is expressed as Equation (1) [36].

(1) $$R={\left|r_{p}\right|}^{2}$$

where

(2) $$r_{p}=\frac{\left(m_{11}+m_{12}q_{n}\right)q_{1}-\left(m_{21}+m_{22}q_{n}\right)}{\left(m_{11}+m_{12}q_{n}\right)q_{1}+\left(m_{21}+m_{22}q_{n}\right)}$$

where rp denotes the reflection coefficient. The given formulas describe the TMM used to simulate light propagation and calculate the reflectivity of a multilayer SPR structure as shown in Equation (2)—the reflection coefficient r_p_ for p-polarized light. The characteristic matrix elements m_11_, m_12_, m_21_, and m_22_, respectively, represent the optical response of all intermediate layers between the prism and SM.

where m*_ij_* is defined as in Equation (3):

(3) $$m_{\mathrm{ij}}=\prod_{k=2}^{N-1}m_{k}=\prod_{k=2}^{N-1}\left[\begin{array}{cc} \cos\beta_{k} & -i\sin\beta_{k}/q_{k}\\ -iq_{k}\sin\beta_{k} & \cos\beta_{k} \end{array}\right]=\left[\begin{array}{cc} m_{11} & m_{12}\\ m_{21} & m_{22} \end{array}\right]$$

Multiply the TMMs of each intermediate layer to obtain the characteristic matrix of the multilayer SPR structure. The elements of the overall transfer matrix are represented by m_11_, m_12_, m_21_, and m_22_. For an N-layer SPR configuration, the total matrix is expressed as the product of the characteristic matrices of each layer. Each layer’s RI, thickness, wavelength, and incident angle determine its phase constant (β_k_) and optical admittance (q_k_) as shown in Equation (4). By considering the contributions of each layer, this matrix formulation makes it possible to calculate the reflectivity and reflection coefficient of the proposed sensor.

(4) $$q_{k}=\sqrt{\frac{\mu_{k}}{\varepsilon_{k}}},\;\cos\theta_{k}=\frac{\sqrt{\varepsilon_{k}-n_{1}^{2}\sin^{2}\theta_{1}}}{\varepsilon_{k}}$$

(5) $$\beta_{k}=\frac{2\pi }{\lambda }n_{k}\cos\theta_{k}\left(z_{k}-z_{k-1}\right)=\frac{2\pi d_{k}}{\lambda }\sqrt{\varepsilon_{k}-n_{1}^{2}\sin^{2}\theta_{1}}$$

The β_k_ phase constant (Equation (5)) characterizes the phase shift light accumulates as it passes through the kth layer and depends on the incident angle, thickness d_k_, wavelength λ, and RI n_k_.

### 2.4. Performance Parameters of the Proposed Sensor

Sensitivity (S), DA, FWHM, and FoM are key metrics used to assess the proposed SPR sensor’s performance. These parameters determine the sensor’s ability to detect minute RI changes caused by various cancer cells. Variations in cancerous cell RI cause a shift in the resonance angle. As cancerous cell RI rises, the local RI increases, and the SPR dip shifts to a higher angle because more molecules interact with the TaSe_2_ surface. The most crucial factor in assessing SPR sensor performance is sensitivity. Equation (6) shows that the resonance angle change (Δθ_res_) relates to a unit change in the analyte medium’s RI (Δn_s_) and is known as sensitivity [35].

(6) $$S=\frac{\Delta \theta_{\mathrm{Res}.}}{\Delta n_{s}}\left({}^{\circ}/\mathrm{RIU}\right)$$

DA represents the sharpness of the SPR resonance curve and indicates the sensor’s ability to precisely determine the resonance angle. It is defined as in Equation (7):

(7) $$\mathrm{DA}=\frac{1}{\mathrm{FWHM}}\;(1/{}^{\circ})$$

The angular width of the SPR reflectance dip between the two incidence angles at which the reflectance reaches the resonance dip’s half-depth level, and is known as the FWHM. It is the halfway point between the corresponding baseline reflectance and the minimum reflectance.

A narrow resonance dip provides higher DA. FWHM is the angular width of the SPR resonance curve measured at half of the R_min_ value. A smaller FWHM indicates a sharper resonance response, reduced energy loss, and improved sensing resolution.

The FoM combines sensitivity and resonance sharpness and is used to evaluate the overall sensing capability by the Equation (8):

*FoM* = *S* × *DA*(/*RIU*) (8)

The field, i.e., TMM, can be used to determine the distribution of electromagnetic fields inside the multilayer SPR configuration as shown in Equations (9)–(12). The incident magnetic field $H_{y}^{\mathrm{inc}}$ *inc* and the reflection coefficient (r_p_) for p-polarized incident light are used to express the electric and magnetic field components of the first medium [37].

(9) $$\left[\begin{array}{c} H_{y1}(z)\\ E_{y1}(z) \end{array}\right]=P_{1}\left(z\right)\left[\begin{array}{c} 1+r_{p}\\ q_{1}\left(1-r_{p}\right) \end{array}\right]H_{y}^{\mathrm{inc}},\;z_{1}\leq z\leq z_{2}$$

The propagation matrix P_1_(z) is given by Equation (10), which explains how an electromagnetic wave moves through the initial layer. The trigonometric terms cos(*β_k_*_*at z*_) and *sin*(*β_k_*_*at z*_) represent the phase variation along each layer’s thickness, while *q*_1_ is associated with the layer’s wave impedance.

where *P*_1_(*z*) is written as:

(10) $$P_{1}\left(z\right)=\left[\begin{array}{cc} \cos(\beta_{k\mathrm{at}z}) & \left(i/q_{1})\sin(\beta_{k\mathrm{at}z}\right)\\ iq_{1}\sin(\beta_{k\mathrm{at}z}) & \cos(\beta_{k\mathrm{at}z}) \end{array}\right]$$

For the subsequent layers (j > 2), including the metallic layers (Ag/Au), the two-dimensional material layer (TaSe_2_), and SM, the electromagnetic field distribution is obtained by extending the same transfer matrix approach:

(11) $$\left[\begin{array}{c} H_{\mathrm{yj}}(z)\\ {-E}_{\mathrm{yj}}(z) \end{array}\right]=P_{j}\left(z=z_{j}+d_{j}\right)\left[\begin{array}{c} 1+r_{p}\\ q_{1}(1-r_{p}) \end{array}\right]H_{y}^{\mathrm{inc}},\;z_{j}\leq z\leq z_{j+1}$$

(12) $$P_{j}\left(z\right)=\left[\begin{array}{cc} \cos(\beta_{k(\mathrm{at}z=z-z_{j})}) & \left(i/q_{j})\sin(\beta_{k(\mathrm{at}z=z-z_{j})}\right)\\ iq_{j}\sin(\beta_{k(\mathrm{at}z=z-z_{j})}) & \cos(\beta_{k(\mathrm{at}z=z-z_{j})}) \end{array}\right]$$

where d_j_ denotes the corresponding layer’s thickness, and q_j_ is its optical admittance. The RI, incident angle, wavelength, and thickness of each layer all affect the phase term *β_k_*_*at z*_. These formulas allow the calculation of the reflection response for assessing sensor performance and provide the electromagnetic field distribution throughout the multilayer SPR structure.

## 3. Results and Discussion

### 3.1. Effect of Optimizing Ag and Au Thickness for the Detection of Cancerous Cells in the Range 1.35–1.40

**Figure 2:**
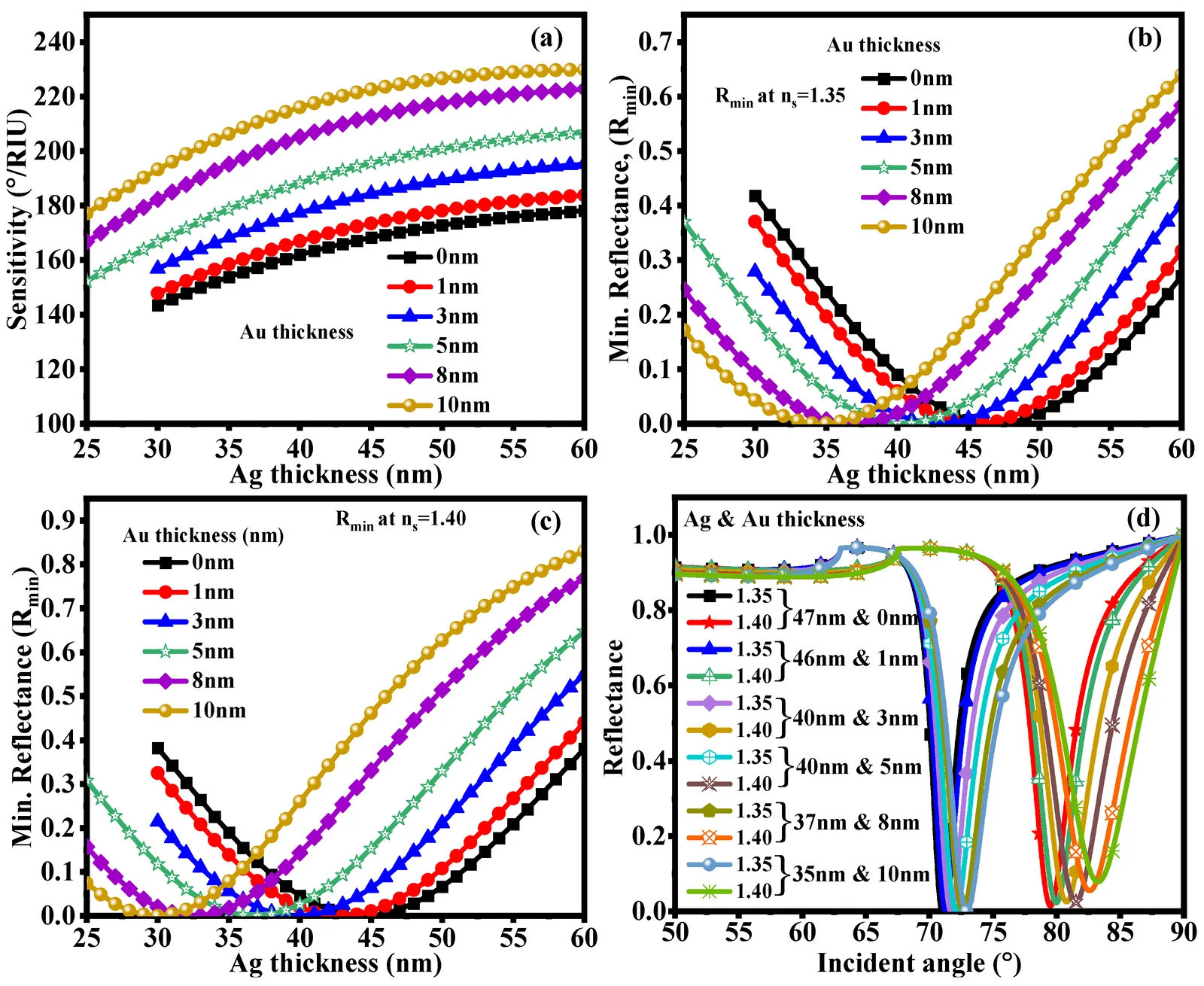
Effect of Au thickness on sensitivity and R_min_ values: (**a**) sensitivity, (**b**) R_min_ at n_s_ = 1.35, (**c**) R_min_ at n_s_ = 1.40, and (**d**) reflectance curve at various Ag and Au thicknesses.

Figure 2 shows how differences in sensitivity and Rmin across various Ag layer thicknesses and Au layer thicknesses (0–10 nm) affect SPR sensing performance. Because it regulates both the electromagnetic field distribution at the metal–analyte interface and the coupling strength between the incident light and SPPs, optimizing the Au thickness is important. Figure 2a shows sensitivity changes with Ag thickness for various Au thicknesses. Because plasmon excitation improves and the interaction between the evanescent field and the analyte medium increases, sensitivity first rises with increasing Ag thickness. The plasmonic field confinement changes, and adding a thin Au layer improves Ag surface stability. Because Au expands the effective sensing region and increases charge-carrier interaction, sensitivity increases significantly as Au thickness rises from 0 to 10 nm. However, an overly thickness Au layer limits molecular interaction and stops further sensitivity improvement by reducing the electromagnetic field’s penetration into the analyte. The sensitivity variation is from 143.40–178.17°/RIU, 147.80–183.75°/RIU, 156.86–195.25°/RIU, 152.24–206.86°/RIU and 170.01–222.68°/RIU with 0 nm, 1 nm, 3 nm, 5 nm, 8 nm and 10 nm Au thickness, respectively. Therefore, maximum sensitivity requires an ideal Au thickness. The non-monotonic variation in Ag thickness and sensitivity results from the interplay between plasmonic coupling and optical damping. For a very thin Ag layer, the interaction between the incident optical field and the surface-plasmon mode is too weak, reducing resonance excitation [38]. Increasing the Ag thickness strengthens the localized electromagnetic field at the metal–dielectric interface and improves momentum coupling, increasing interaction with the SM. However, increased metal volume reduces evanescent-field penetration into the analyte region and adds absorption and damping losses beyond the optimum thickness. As a result, the sensitivity decreases as a smaller fraction of the enhanced field interacts with the SM. Thus, the ideal Ag thickness represents a compromise between field penetration, metallic loss, and efficient plasmon excitation. The Rmin value indicates the efficiency of energy transfer from incident light to the SPW and also reflects the depth of the SPR resonance dip. Stronger plasmon excitation and improved resonance coupling are correlated with lower R_min_ values. Figure 2b shows that, for an analyte RI of n_s_ = 1.35, R_min_ first drops as Ag thickness increases before reaching a minimum at an optimal Ag thickness. This decrease suggests that the incident optical wave and the SP mode couple effectively. R_min_ values rise above the ideal Ag thickness because the metal layer becomes thicker than the electromagnetic field’s effective PD, increasing optical losses and reducing analyte interaction. Figure 2c similarly shows the variation in R_min_ at a higher analyte RI (n_s_ = 1.40). The increased RI of the SM shifts the resonance behavior. The dielectric environment affects the plasmon wave-vector matching condition, causing a slight change in the optimized Ag thickness. A lower Rmin value indicates a sharper, stronger SPR response, enhancing resonance resolution and DA. Au thickness crucially balances sensitivity, PD, and field confinement. A thin Au layer maintains strong plasmon coupling and chemical stability, while an excessively thick Au layer increases damping losses. The optimized Ag/Au thickness combination produces strong SPR signals with high sensitivity, low Rmin values, and enhanced sensing performance for RI-based detection. Figure 2d shows the reflectance curves of the proposed hybrid sensor for various bimetal and TaSe_2_ thickness combinations. To maximize the plasmonic layer thickness and improve sensing performance, we examine the relationship between incident angle and reflectance. The resonance angle (θ_res_), where incident light energy is effectively transferred to the SPP at the metal–dielectric interface, is indicated by the sharp dip in the reflectance curves. The thickness of the Ag, Au, and TaSe_2_ layers strongly affects the location and depth of this resonance dip. The TaSe_2_ layer affects the electromagnetic field confinement and interaction with the SM, whereas variations in Ag thickness alter the plasmon excitation efficiency. The deeper, sharper Rmin value from the optimized Ag/TaSe_2_ thickness combination indicates stronger plasmon coupling and lower optical losses. While a narrow resonance width denotes better DA, a lower R_min_ value indicates effective energy transfer from the incident wave to the SP mode. The resonance dip shifts toward higher incident angles as the SM’s RI rises because the dielectric environment near the sensing interface changes. The resonance angle of 71°, 71.24°, 71.68°, 72.08°, 72.62° and 72.93° for RI of 1.35 and 79.51°, 79.96°, 80.77°, 81.50°, 82.60° and 83.24° for RI of 1.40 is obtained with 47 nm & 0 nm, 46 nm & 1 nm, 43 nm & 3 nm, 40 nm & 5 nm, 37 nm & 8 nm and 35 & 10 nm Ag & Au thickness, respectively. The findings show that Ag and TaSe_2_ layer thickness significantly influences resonance behavior. An overly thick Ag layer increases damping losses and reduces field penetration into the analyte region, while an overly thin Ag layer weakens plasmon excitation. Similarly, increasing Au thickness increases electromagnetic field amplification and molecular interaction, improving sensitivity. Thus, the optimized bimetal/TaSe_2_ configuration is appropriate for precise biological cell detection because it offers a balanced combination of high sensitivity, low Rmin, and a sharp SPR response. Table 3 shows the optimized bimetallic layer thickness and its impact on SPR sensor performance. The findings show that increasing Au thickness from 0 to 10 nm increases sensitivity from 170.26 to 206.32°/RIU due to better analyte interaction and improved electromagnetic field confinement. The optimal Ag (35 nm)/Au (10 nm) configuration shows stronger plasmonic coupling, delivering the highest sensitivity, along with a lower FWHM and better DA. The variation in R_min_ and FWHM demonstrates how important Ag/Au thickness is in regulating sensing performance and resonance sharpness. During the thickness sweep, the highest sensitivity achieved is 222.68°/RIU under the specified sweep condition. In contrast, the selected 35 nm Ag/10 nm Au configuration used for the subsequent performance analysis has a sensitivity of 206.32°/RIU. These values correspond to different optimization conditions.

### 3.2. Effect of Ag Thickness on Sensitivity and Rmin Value for Various Cancerous Cell RIs

**Figure 3:**
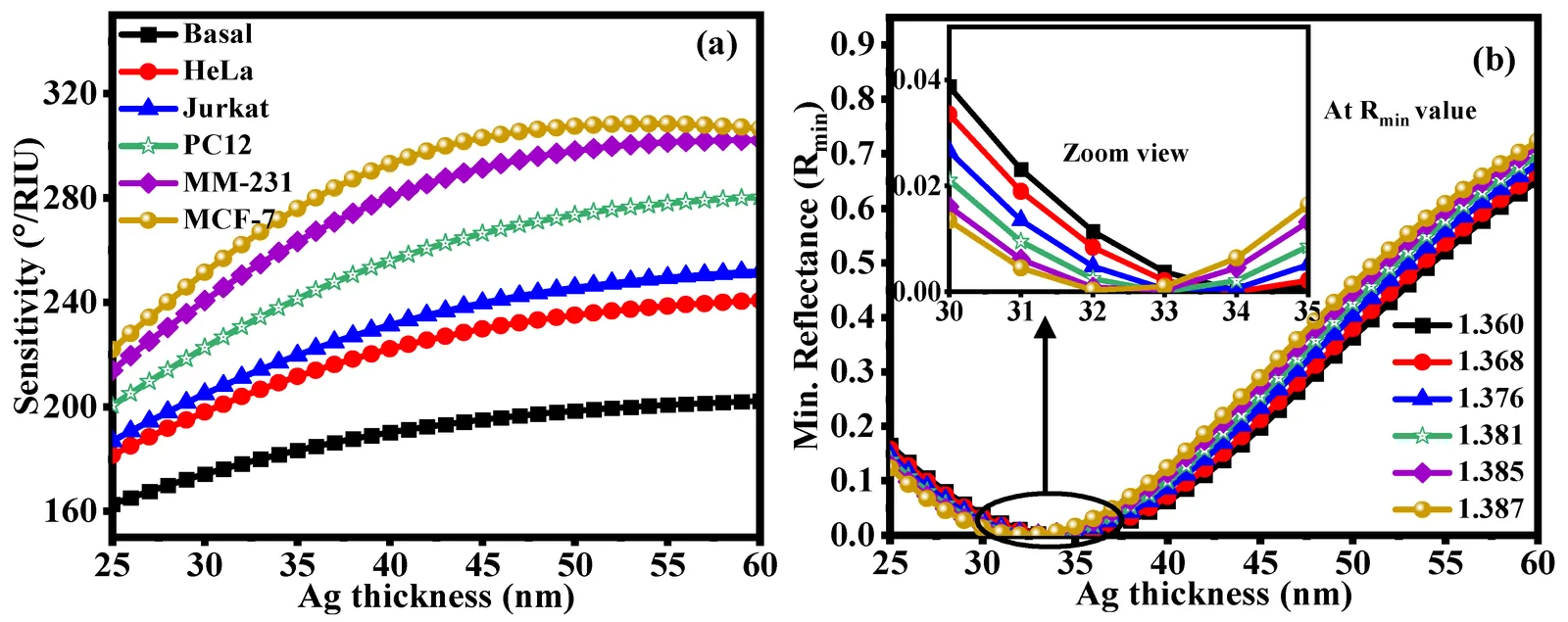

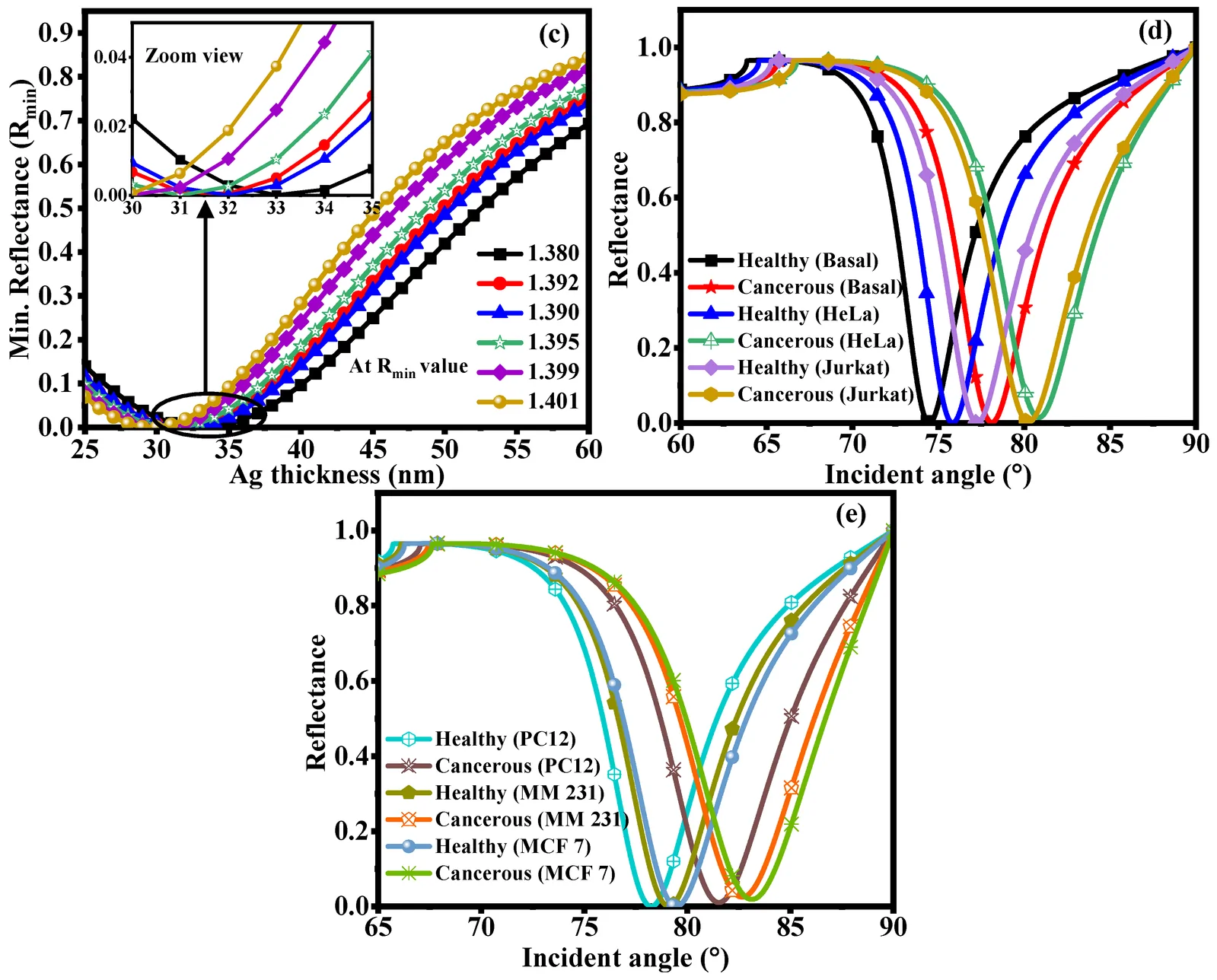
Optimization of Ag layer thickness for the proposed SPR sensor: (**a**) sensitivity variation for different biological sensing layers, (**b**) R_min_ variation at different analyte RIs (n_s_ = 1.360–1.387), and (**c**) R_min_ variation for higher RI range (n_s_ = 1.380–1.401). Insets show the magnified view of the SPR resonance region near the optimized Ag thickness (**d**,**e**) reflectance curve at various healthy and cancerous cells.

Depending on the analysis goal, we optimized Ag thickness in two separate phases. To achieve an appropriate bimetallic plasmonic configuration across the examined refractive-index range, firstly optimized the Ag thickness and Au overlayer. This analysis yielded 35 nm Ag and 10 nm Au as the representative optimized bimetallic structure. To determine the corresponding maximum RI sensitivity for biological sensing, we then independently varied the Ag thickness for each SM. As a result, the nominal 35 nm Ag thickness used in the broad-range bimetallic analysis may not match the ideal Ag thickness found for a particular biological sensing condition. Figure 3 shows how Ag layer thickness affects the sensing performance of the proposed SPR structure by analyzing variations in sensitivity and R_min_. Because the plasmonic metal layer thickness determines the coupling efficiency between incident light and the SPP mode at the metal–dielectric interface, optimizing Ag thickness is essential. Figure 3a shows the sensitivity variation as a function of Ag thickness for various dielectric layer materials, including Basal, HeLa, Jurkat, PC12, MM-231, and MCF-7. Sensitivity rises steadily as Ag thickness increases up to a certain point before approaching saturation. Improved confinement of the electromagnetic field near the metal–analyte interface, which enhances interaction between the evanescent field and the SM, drives the increased sensitivity at higher Ag thickness. Because these biological layers have varying RI values, MCF-7 shows the highest sensitivity among the dielectric layers examined, followed by MM-231, PC12, Jurkat, HeLa, and Basal. The SPR resonance position shifts more strongly when the RI contrast between the metal surface and the SM is higher, which increases sensitivity. Figure 3b shows how R_min_ varies with Ag thickness for various analyte RIs (n_s_ = 1.360–1.387). The R_min_ value indicates the strength of optical energy transfer from the incident wave to the surface plasmon mode and also reflects the depth of the SPR resonance dip. Stronger resonance coupling, lower reflected optical power, and better detection accuracy all correlate with lower R_min_ values. Reflectance first decreases as Ag thickness increases and reaches a minimum at an ideal Ag thickness (roughly 30–33 nm), indicating maximum plasmon excitation. R_min_ rises above this ideal thickness because a thicker Ag layer reduces electromagnetic-field penetration into the analyte region and adds more optical damping. Figure 3b shows the inset and enlarged views of the resonance region, making it clear how Rmin varies near the optimal Ag thickness. Figure 3c shows the variation in R_min_ for another range of analyte RIs (n_s_ = 1.380–1.401). As the RI increases, the resonance condition shifts because the dielectric environment at the metal–analyte interface changes. The SPR dip shifts toward higher Ag thickness, confirming that plasmon coupling depends on the RI of the SM. The zoomed inset highlights minor variations in R_min_ around the optimized region, which are important for assessing resonance sharpness and DA. Overall, Figure 3 shows that Ag thickness significantly affects the SPR sensor’s sensitivity and resonance properties. An optimized Ag layer balances strong plasmon excitation, low optical loss, narrow resonance width, and improved interaction with the SM. Therefore, in biological and RI-based sensing applications, choosing an appropriate Ag thickness is essential for obtaining high sensitivity and an improved FOM. Figure 3d shows the reflectance curves for various healthy and cancerous cell lines at the sensing interface. Because different cell types have different RIs, the momentum-matching condition between the incident light and the SPW changes, resulting in variations in the resonance angle. Plotting the reflectance intensity against the incident angle (°) is shown in Figure 3d,e. Each curve represents a distinct biological cell type, such as healthy and cancerous cells. The resonance angle (θ_res_), where the maximum energy transfers from the incident light to the surface plasmon polariton at the metal–analyte interface, is represented by the sharp dip in each curve. The resonance is obtained of 74.47° for Healthy (Basal), 75.82° Healthy (HeLa), 77.82° Healthy (Jurkat), 78.25° Healthy (PC12), 79.10° Healthy (MM 231) and 79.46° Healthy (MCF 7) and 78.10° Cancerous (Basal), 80.84° Cancerous (HeLa), 80.25° Cancerous (Jurkat), 81.53° Cancerous (PC12), 82.67° Cancerous (MM 231) and 83.13° Cancerous (MCF 7). The resonance angle shifts by 3.63°, 5.01°, 2.99°, 3.28°, 3.56°, and 3.66° for skin, cervical, blood, Adrenal gland, Breast I, and Breast II cancer. Strong plasmon excitation causes the reflected light intensity to drop to its lowest value at this point. As the RI of the SM increases, the resonance positions tend to shift towards higher incident angles. Cancerous cells have higher RIs due to increased cellular density, altered morphology, and higher intracellular biomolecular concentration. In contrast, healthy cells typically exhibit lower RIs and therefore show resonance at comparatively smaller angles. As a result, cancerous cell lines show better discrimination by producing a larger shift in the resonance angle. Table 4 shows the optimized sensing performance of the suggested SPR sensor for identifying various healthy and cancerous cells. For every analyte, the Ag layer thickness was adjusted to maximize sensitivity and enhance resonance properties. The findings show that sensitivity increases as cellular RI increases. At an optimized Ag thickness of 32 nm, MCF-7 shows the highest sensitivity (262°/RIU), followed by MM-231, PC12, Jurkat, HeLa, and Basal cells. The lower Rmin values and narrow FWHM obtained for various cell lines indicate strong plasmon excitation, increased DA, and improved ability to distinguish between healthy and malignant cells. Moreover, Table 4 shows the maximum sensitivity at the optimized Ag thickness and sensing performance parameters for various biological cells. The findings show that sensitivity increases with the SM’s RI, with Jurkat and MCF-7 achieving a maximum sensitivity of 290.24°/RIU at an ideal Ag thickness of 39 nm. These cell lines have the lowest R_min_ values, which suggest better detection capabilities, sharper resonance, and stronger SPR excitation. The broad RI analysis of the chosen Ag/Au bimetallic configuration yields a sensitivity of about 206°/RIU. In contrast, the biological sensing analysis with its corresponding optimized conditions yields a sensitivity of about 262°/RIU. The highest sensitivity attained for a specific biological sensing medium, after independent Ag thickness optimization, is roughly 290°/RIU. As a result, these sensitivity values should be interpreted as performance values for a single fixed sensor configuration. Rather, they show how the SM, Ag thickness, and optimization conditions all affect the theoretical sensitivity. The highest sensitivity does not always translate into the best overall sensing performance, even though sensitivity is a crucial factor in assessing an SPR sensor. A thorough evaluation should consider sensitivity alongside Rmin, FWHM, DA, and FoM. While a higher FoM denotes a good balance between sensitivity and resonance width, a narrow FWHM denotes a sharper resonance and better angular resolution. Consequently, a wider resonance width combined with increased sensitivity may not always improve overall sensor performance. As a result, this study assesses Ag thickness using a variety of sensing parameters. It selects the best configuration by considering the combined behavior of sensitivity, resonance sharpness, DA, and FoM rather than sensitivity alone. Independent numerical optimization was used to determine the Ag thickness values shown in Table 5 for each sensing condition considered. Therefore, rather than a single fixed sensor optimized simultaneously for all cancer types, the results show optimization of the suggested sensor structure based on analyte or RI conditions. The SPR response and sensitivity of the suggested structure are examined in relation to changes in the effective RI using the various optimized Ag thicknesses. Practical multi-cancer identification would require a fixed device configuration, selective recognition components, multiplexed sensing channels, or other target-specific mechanisms.

### 3.3. Compare to Conventional Structure

**Figure 4:**
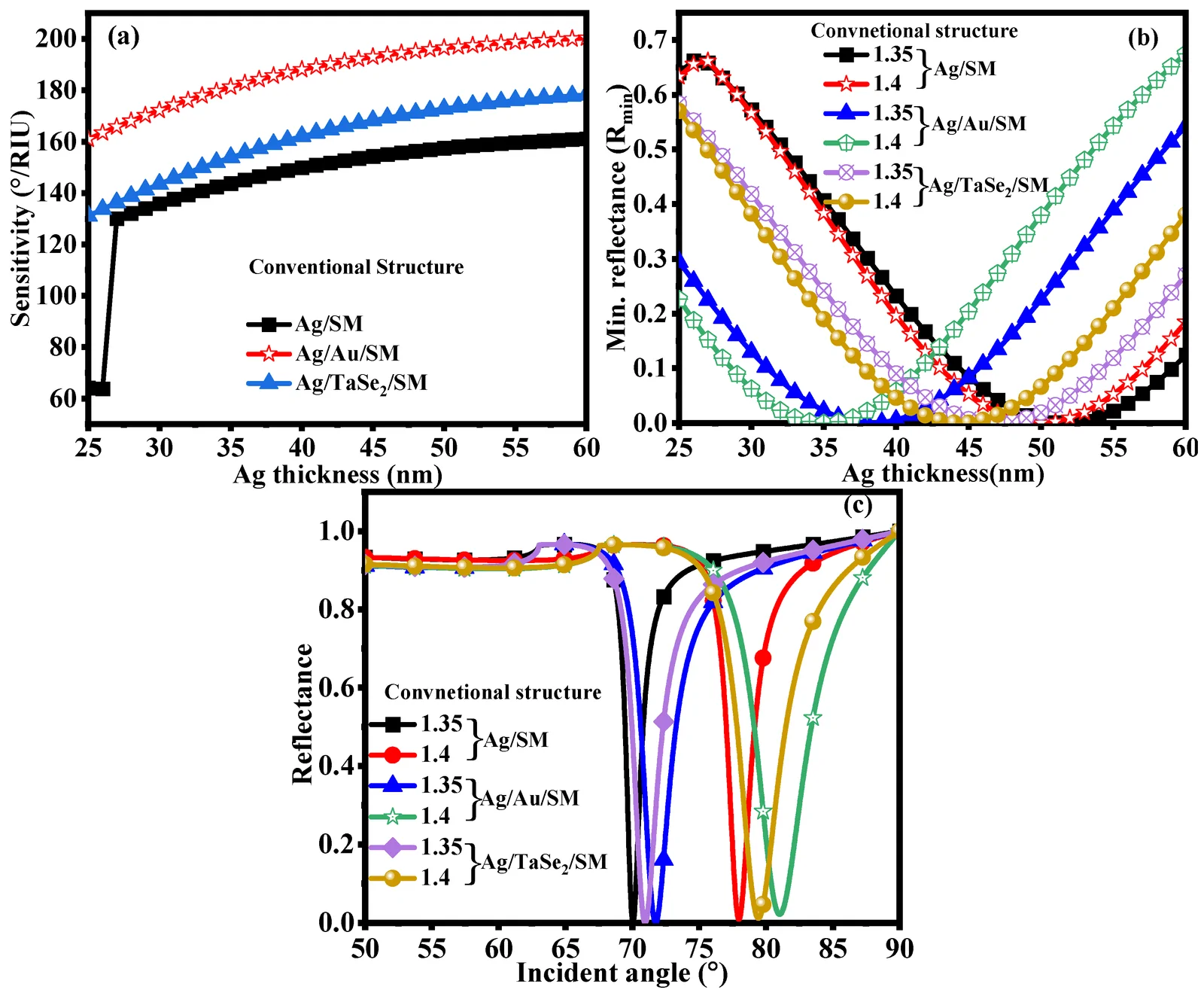
Optimization of Ag layer thickness for the conventional SPR sensor Ag/SM, Ag/Au/SM and Ag/TaSe_2_/SM: (**a**) sensitivity variation, (**b**) R_min_ variation at n_s_ = 1.335–1.40, and (**c**) reflectance curve with RIs (n_s_ = 1.335–1.40).

Compare the proposed Ag/Au/TaSe_2_ configuration with corresponding Ag-only, Ag/Au, and Ag/TaSe_2_ structures under identical simulation conditions to quantify the contribution of each sensing layer. All configurations should adhere to the same BK7 prism, operating wavelength, analyte RI, and evaluation procedure. Include resonance angle, sensitivity, R_min_, FWHM, DA, FoM, and sensitivity in the comparison. By comparing the individual contributions of the Au protective layer and TaSe_2_ sensing layer to the hybrid structure’s overall response, this controlled comparison enables differentiation. The Ag layer primarily drives efficient surface-plasmon excitation; the Au layer is more chemically stable and alters plasmonic damping; and the TaSe_2_ layer is more accessible at the sensing interface for local electromagnetic interaction and analyte access. Figure 4 analyzes the optical and sensing performance of the conventional Ag/SM, Ag/Au/SM, and Ag/TaSe_2_/SM SPR configurations by examining the effect of Ag-layer thickness. Figure 4a shows that sensitivity varies significantly with Ag thickness within the investigated range of approximately 25 to 60 nm. The sensitivity of the conventional Ag/SM structure initially rises rapidly from approximately 64.11°/RIU at 25 nm. However, as Ag thickness increases, the sensitivity gradually rises to approximately 160.10°/RIU at 60 nm. Across the entire thickness range, the Ag/Au/SM structure shows significantly higher sensitivity, increasing from approximately 160.95°/RIU to nearly 200.31°/RIU. Compared with the conventional structure, the Ag/TaSe_2_/SM configuration shows a more robust sensing response, increasing sensitivity from approximately 131.02°/RIU to approximately 178.17°/RIU. These results indicate that the RI-dependent response is enhanced and that incorporating Au and TaSe_2_ alters the optical interaction at the metal–analyte interface. Changes in the SP excitation condition, electromagnetic-field confinement, and the interaction between the evanescent field and the SM are all linked to the sensitivity enhancement. Figure 4b further shows how Rmin varies with Ag thickness, ranging from approximately 1.35 to 1.40 for different analyte RIs. The Ag layer thickness strongly affects the coupling efficiency between the surface plasmon mode and the incident optical field, as shown by the systematic shift in Rmin with Ag thickness and refractive index. Stronger energy transfer from the incident beam to the surface plasmon mode is associated with a lower R_min_, which in turn results in a more pronounced SPR resonance. Adding Au or TaSe_2_ also changes the resonance condition and optical losses of the multilayer structure, as shown by the different curves for the Ag/SM, Ag/Au/SM, and Ag/TaSe_2_/SM configurations. The TaSe_2_ layer introduces additional interaction at the metal–dielectric interface, resulting in different reflectance characteristics, while the Au layer modifies the effective optical environment surrounding the Ag layer. Figure 4c shows the corresponding reflectance curve as a function of incident angle for different RIs. The SP mode is excited, as shown by the distinct, well-defined resonance dip in each sensing configuration. The resonance dip shifts toward a higher incident angle as the SM’s RI increases. This is the fundamental sensing mechanism employed for RI detection. A larger angular shift for a given change in RI indicates higher angular sensitivity. Therefore, the observed shift in resonance position for the investigated structures confirms their ability to distinguish small variations in the SM’s RI. Figure 4c shows similar results. The third illustration shows that Ag thickness plays a crucial role in the design, influencing sensitivity, R_min_, resonance position, and plasmonic coupling strength simultaneously. The comparison of the three configurations also shows that the modified Ag/Au/SM and Ag/TaSe_2_/SM structures are more sensitive than the conventional Ag/SM configuration, with the Ag/Au/SM structure having the highest sensitivity in the Ag-thickness range studied. These results show that the Ag-layer thickness must be optimized before choosing the final multilayer SPR sensor configuration, because an appropriate thickness can generate the most effective SP excitation and the most distinct resonance response. Table 6 compares the optimized performance of the Ag/SM, Ag/Au/SM, and Ag/TaSe_2_/SM structures for ns = 1.33–1.40. The optimized Ag thicknesses are 52, 38, and 47 nm, respectively. The Ag/Au/SM structure offers the highest sensitivity (185.58°/RIU), followed by Ag/TaSe_2_/SM (170.26°/RIU) and Ag/SM (158.43°/RIU). Nevertheless, Ag/SM shows the highest detection accuracy (117.35/°) and FoM (117.35/RIU), whereas Ag/Au/SM and Ag/TaSe_2_/SM show FoM values of 70.29 and 73.07/RIU, respectively. These findings suggest that adding Au or TaSe_2_ enhances sensitivity while also altering the FWHM and DA.

### 3.4. Analysis of Normalized Electric Field Normalized and SPP Mode

**Figure 5:**
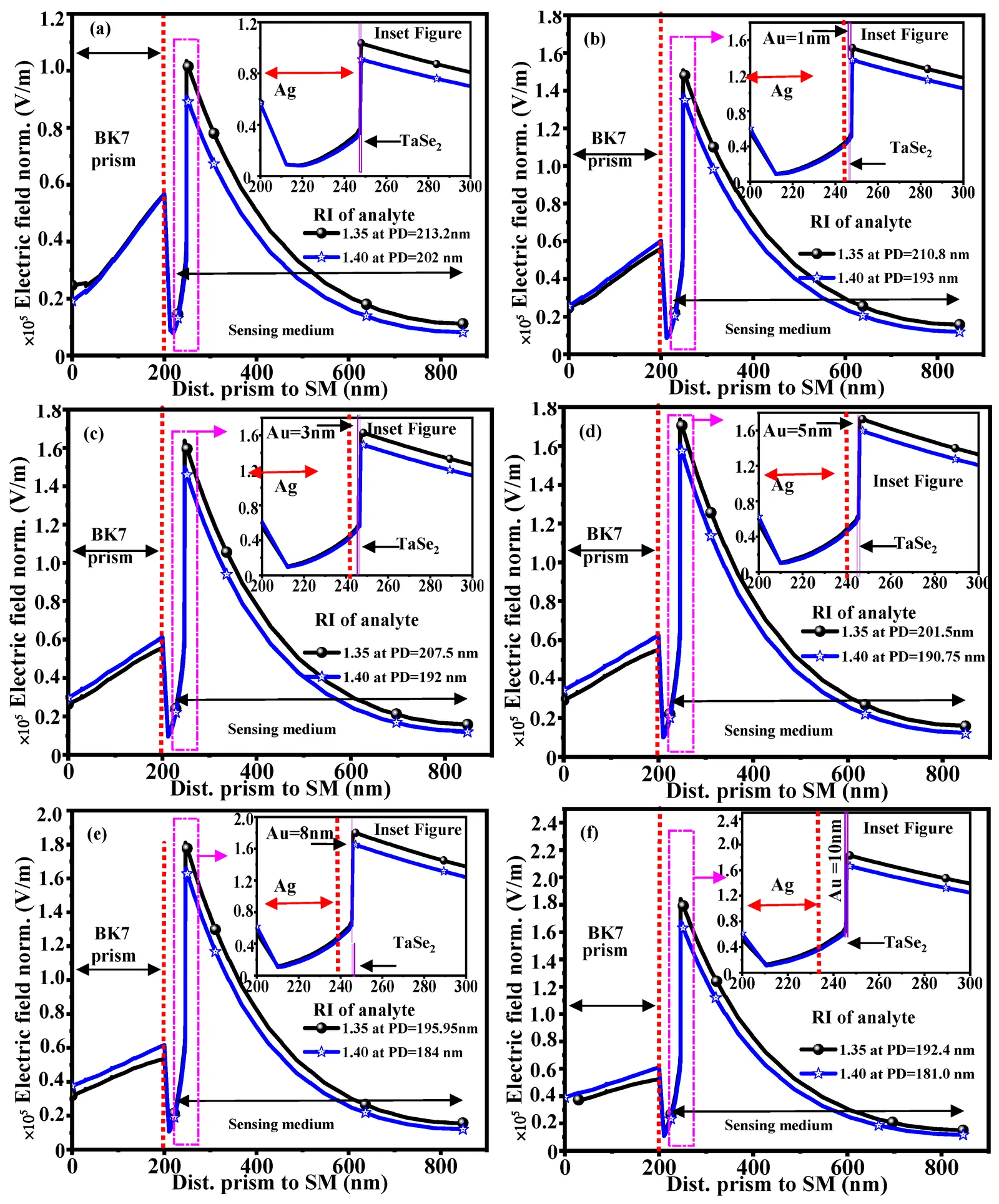
Electric field and PD analysis with various Au thickness: (**a**) Au = 0 nm, (**b**) Au = 1 nm, (**c**) Au = 3 nm, (**d**) Au = 5 nm, (**e**) Au = 8 nm, and (**f**) Au = 10 nm.

Figure 5 shows the normalized electric field and field-confinement properties of the suggested SPR structure. When assessing the strength of the interaction between the excited SPW and the SM, the electric field distribution is an important factor. At the metal–dielectric interface in SPR sensors, incident p-polarized light generates an evanescent electromagnetic field that exponentially decays away from the interface and into the SM. The effective PD of this evanescent field, where the electric field intensity drops to about 37% of its maximum value at the interface, is calculated using the 1/e decay factor. The PD is calculated by the |*E*(*z*)/*E*(0)| = 1/*e*, where (E(0)) represents the electric-field amplitude at the sensing interface and (E(z)) represents the field amplitude at a distance (z) into the SM. The PDs at 192.4 nm and 181 nm are obtained at RI SM of 1.36 and 1.40, respectively. This PD indicates the effective sensing region where analyte molecules or biological cells interact with the enhanced electromagnetic field. The electric-field normalized (norm.) varies with Au-layer thickness in the SPR structure, as illustrated in Figure 5. There are six cases in the figure, indicating (a) Au = 0 nm, (b) Au = 1 nm, (c) Au = 3 nm, (d) Au = 5 nm, (e) Au = 8 nm, and (f) Au = 10 nm. In each scenario, the electric-field response is shown by plotting the distance from the BK7 prism, through the metallic layers, and into the SM for various analyte RIs. The excitation of the SP mode is indicated by the sharp increase in the electric field near the Ag/Au/TaSe_2_–SM interface. The evanescent nature of the SPR field is shown by the strong localization of field intensity near the interface and its gradual decrease as distance increases inside the SM. Figure 5a, which corresponds to a 1 nm Au thickness, shows a pronounced enhancement in the electric field near the metal–SM interface. The field gradually decreases as it approaches the interface, indicating strong surface confinement. The inset shows the multilayer region in a larger view and clearly shows the Ag, Au, and TaSe_2_ layers. Figure 5b shows the effect of increasing the Au thickness to 1 nm; the electric-field distribution changes significantly, modifying both the field enhancement and penetration profile due to the added Au layer. The localized plasmonic field is sensitive to changes in the SM of RI, as evidenced by the slightly different field distributions produced by the two analyte RI conditions. Near the sensing interface, Au = 3 nm exhibits a stronger and more extended electric-field response, as seen in Figure 5c. This implies that Au thickness is a major factor in coupling the incident optical field to the surface plasmon mode. The corresponding inset provides an expanded view of the field behavior in the Ag/Au/TaSe_2_ region. The increased metallic thickness further modifies the electric-field distribution, but it remains strongly concentrated near the interface in Figure 5d, where the Au thickness is 5 nm. Only a limited region near the interface contributes strongly to SPR sensing, as the field gradually decays toward the SM. Likewise, Figure 5e shows the field distribution for 8 nm Au thickness, with a gradual reduction in field magnitude with distance and pronounced field localization at the sensing interface. The sensing medium’s optical properties affect the electric field, as shown by the differences in responses at the two RIs. Finally, the largest Au thickness studied is 10 nm, as shown in Figure 5f (proposed structure). After a gradual decay into the sensing region, the electric-field profile is again strongly enhanced around the metal–SM interface. The inset clearly shows the individual Ag, Au, and TaSe_2_ layers and illustrates their contribution to the near-interface field distribution. The interaction between the incident optical wave and the collective oscillation of free electrons in the Ag/Au bimetallic layer drives the strong field localization at this interface. Because of its high electrical conductivity and negative real part of the dielectric permittivity, the Ag layer strongly excites plasmons. In contrast, the Au layer enhances metallic surface stability and sustains effective plasmon propagation. Because of its high carrier mobility, strong light-matter interaction, and abundance of active surface sites, the ultrathin TaSe_2_ layer further increases electric-field intensity. The interaction between the evanescent field and analyte molecules is enhanced because electromagnetic energy is more effectively confined near the sensing region. Figure 5f shows an enlarged view of the electric field distribution within individual layers, such as Ag, Au, and TaSe_2_. The field intensity gradually diminishes inside the SM and is primarily concentrated around the metal–2D material interface. A substantial amount of the enhanced electromagnetic field extends into the SM, as confirmed by the PD determined by the 1/e decay criterion. This enables effective detection of RI variations brought on by biological cells or analyte molecules. Changes in the SM’s RI alter the plasmon wave-vector matching condition, which alters the PD and field distribution.

**Figure 6:**
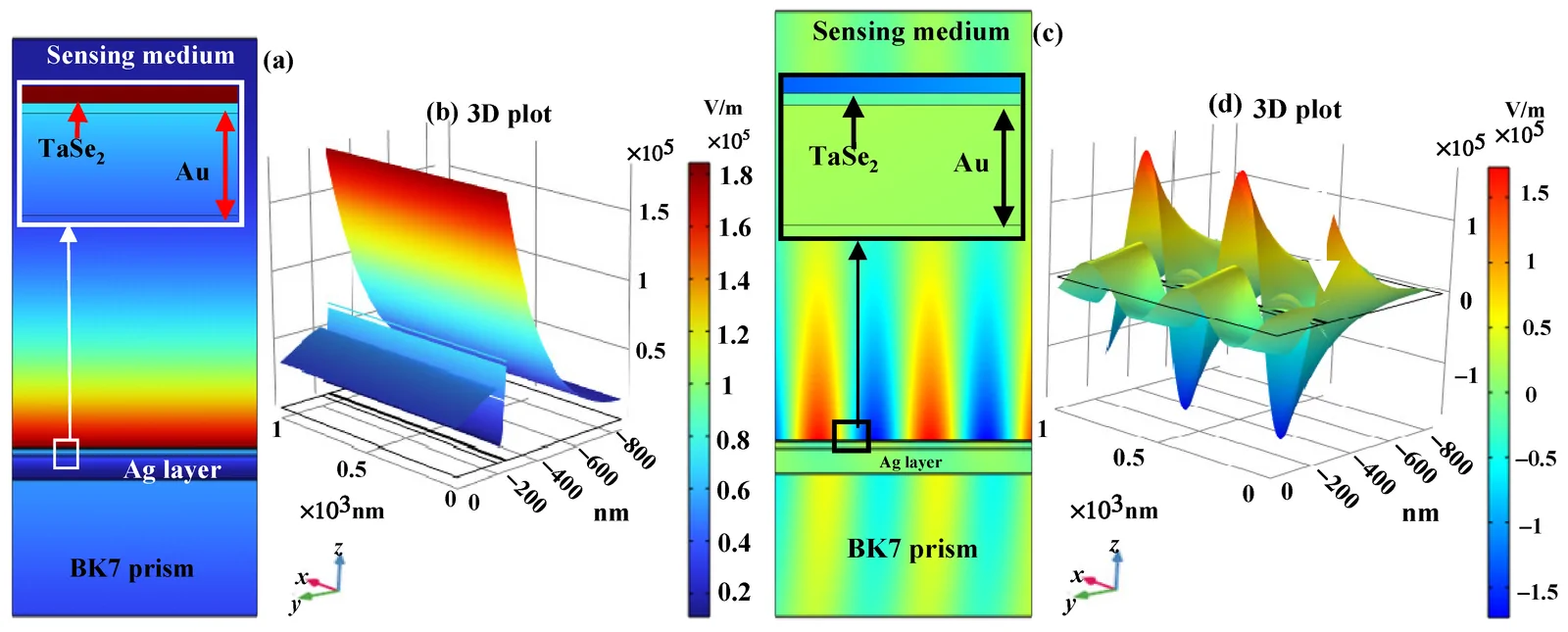
(**a**,**b**) 2D and 3D electric field distribution, and (**c**,**d**) 2D and 3D SPP mode.

The optimized SPR structure’s 2D electric field intensity in Figure 6a,d shows that the electromagnetic field is spatially localized near the sensing interface, corresponding to the 3D field distributions shown in the diagrams. Figure 6c,d confirm the strong concentration of electromagnetic energy in the Ag, Au, and TaSe_2_ layers and provide a detailed visualization of the enhanced field. The high-intensity localization indicates effective plasmonic excitation and increased coupling between the optical wave and surface plasmon polaritons. This enhanced field confinement results in a larger SPR shift and increased sensitivity, increasing the probability of interaction between the sensing layer and the target molecules. According to the electric-field analysis, the optimized Ag, Au, and TaSe_2_ hybrid configuration offers strong electromagnetic performance and a suitable penetration depth for sensor applications. For biological and IR sensing applications, adding TaSe_2_ significantly improves spatial localization and analyte interaction, increasing sensitivity, improving DA, and enhancing the performance of the proposed SPR.

### 3.5. Fabrication, Stability, Repeatability and Error Feasibility of the Proposed Structure

**Figure 7:**
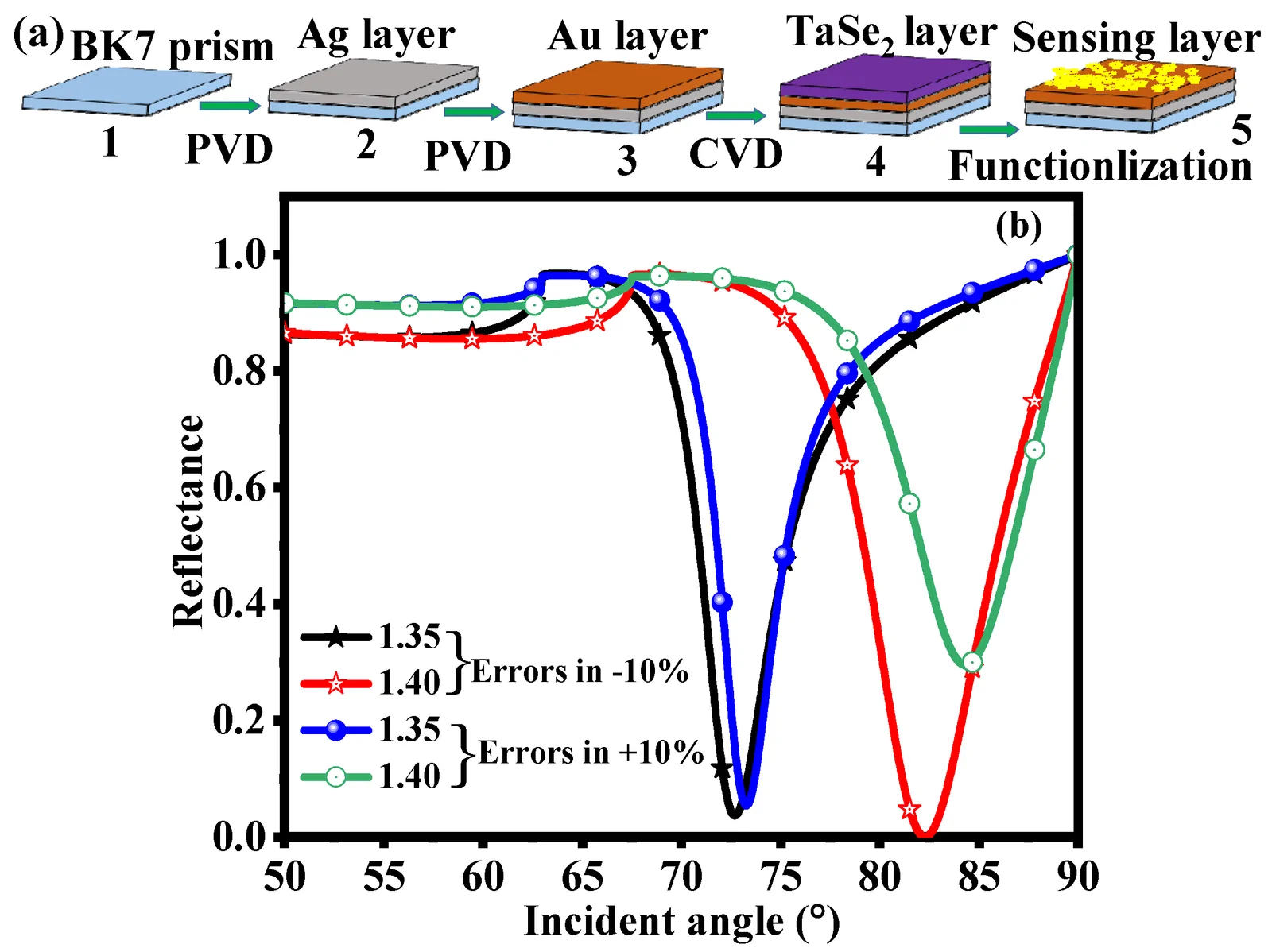
(**a**) Schematic fabrication process and (**b**) reflectance response under ±10% variations in the constituent-layer thicknesses.

Figure 7a shows the schematic fabrication process for the proposed configuration. Well-known thin-layer deposition and 2D material transfer methods can be used to create the multilayer sensor. Because of its excellent optical transparency and appropriate RI, a clean BK7 glass prism is first utilized as the optical coupling substrate [39,40]. To remove organic impurities and improve surface adhesion, the prism surface is first cleaned using conventional chemical and plasma treatment techniques. Next, a Physical Vapor Deposition (PVD) method, such as thermal evaporation or electron-beam evaporation, is used to deposit a thin layer of Ag onto the BK7 prism. Because of its excellent optical qualities and high electrical conductivity, the optimized Ag thickness offers effective SP excitation [27]. A thin Au layer is deposited over the Ag layer to address Ag oxidation. The Au layer maintains long-term plasmonic performance while preserving effective electromagnetic coupling, enhancing chemical stability, and preventing Ag surface deterioration [27]. Then, using methods like mechanical exfoliation, liquid-phase exfoliation, or Chemical Vapor Deposition (CVD), an ultrathin TaSe_2_ layer is transferred or deposited onto the Au surface [33]. Because of its large surface area, strong light-matter interaction, superior charge-transport characteristics, and abundant active sites for analyte adsorption, the TaSe_2_ layer serves as the active sensing interface. The final fabricated structure consists of a functionalized TaSe_2_ surface exposed to the sensing medium, where biological cells or target analyte molecules interact with the 2D material layer. Analyte adsorption produces a detectable SPR shift by altering the local RI near the sensing interface. In terms of stability, the Ag/Au bimetallic configuration offers better environmental durability than a single Ag layer. The Au coating shields Ag from oxidation and corrosion while maintaining strong plasmonic excitation. Because of its strong layered structure and chemical resistance, the TaSe_2_ layer improves structural stability. Strong adhesion between the prism substrate and the deposited layers reduces delamination and ensures dependable sensor performance over time. The suggested sensor’s repeatability can be achieved through consistent surface functionalization techniques, uniform deposition methods, and precise control of layer thickness. The well-defined multilayer structure ensures reproducible plasmonic coupling conditions, leading to consistent resonance angle shifts for identical analyte concentrations. Regenerating the TaSe_2_ sensing surface enables multiple sensing cycles without significantly degrading the SPR response. As a result, the proposed structure is suitable for realistic label-free biosensing applications because of its high measurement repeatability, enhanced chemical stability, and excellent fabrication feasibility. Surface roughness is characterized experimentally or through advanced numerical methods. For the proposed multilayer structure, it is important to control the Ag and Au thicknesses with precision, because small deviations can change the plasmonic phase-matching condition and resonance linewidth. A controlled transfer or deposition process is required to obtain a uniform ultrathin TaSe_2_ layer over the Au surface. Thickness non-uniformity can alter the effective optical path and local field confinement, while surface roughness can cause additional scattering and broaden the SPR resonance. Therefore, carefully control surface morphology, adhesion, and uniformity during fabrication.

Figure 7b shows the impact of a ±10% change in the thickness of the constituent layers on the sensing parameters and the reflectance curve for two analyte RIs (n_s_ = 1.35 and 1.40). The resonance dip shifts with changes in sensing conditions, confirming the suggested SPR structure’s sensitivity to RI variations. The resonance angle and R_min_ value change slightly when the parameters are slightly off, but the overall SPR response stays constant. The maintained resonance properties show that the suggested sensor has good fabrication tolerance and is resilient to potential experimental errors. Table 7 shows the impact of ±10% thickness variation in each constituent layer on the suggested SPR sensor’s performance. The findings show that Sensitivity, R_min_, FWHM, DA, and FoM vary slightly with small changes in Ag, Au, and TaSe_2_ thickness because of changes in plasmon coupling conditions. The sensitivity exhibits good tolerance against fabrication uncertainties, ranging from 191.68 to 221.16°/RIU. The sensor’s robustness and practical fabrication feasibility are confirmed by its stable SPR characteristics despite thickness errors that affect resonance sharpness and FoM. Even with potential layer-thickness variations, the optimized structure performs satisfactorily.

While the suggested Ag/Au/TaSe_2_ configuration shows improved electromagnetic-field confinement and RI sensitivity, the current model does not describe molecular recognition or cancer-specific biomarker binding. Because the simulations use the SM’s effective RI values, they show how the suggested structure can detect RI variations rather than a specific cancer biomarker. Characterization of biomarker binding, selectivity, response in complex biological media, and experimental SPR response would follow once the TaSe_2_ sensing interface is experimentally functionalized with a suitable biorecognition element for selective cancer detection. Later experimental studies will address these factors, as they are outside the scope of the current theoretical investigation. Material stability alone does not ensure a stable baseline during repeated measurements for practical biosensing. Proteins and other biomolecules can undergo non-specific adsorption, altering the local refractive index and causing resonance shifts unrelated to the target analyte. Replication can be reduced by baseline drift, surface contamination, and residual sample components. Regeneration protocols, controlled washing procedures, anti-fouling strategies, and appropriate surface functionalization can all help mitigate these effects. The current theoretical model does not explicitly include biomolecular fouling, baseline drift, regeneration cycles, or time-dependent degradation. Therefore, future studies should include repeated sensing and regeneration cycles under representative biological conditions to evaluate regeneration performance and long-term stability experimentally.

### 3.6. Limitations of the Present Work

To examine the optical response and sensing performance of the proposed configuration, this study mainly relies on a theoretical TMM. Although the numerical results show promising RI sensitivity and advantageous SPR properties, several limitations remain. First, experimental validation is necessary because the proposed structure has not yet been fabricated and characterized. Variations in metal and TaSe_2_ thickness, surface roughness, adhesion, film morphology, deposition conditions, and material Optical constants may affect the SPR response in a practical device. Second, the theoretical model’s optical constants may not match those of experimentally deposited thin layers because they represent idealized or literature-based material parameters. Third, the current model does not simulate analyte-specific molecular recognition, adsorption kinetics, biochemical binding, or surface functionalization; instead, it mainly accounts for RI variation in the SM. Therefore, rather than being direct experimental proof of selective cancer biomarker detection, the reported results should be understood as theoretical predictions of RI-based sensing performance. To confirm the anticipated sensitivity, stability, repeatability, and selectivity of the suggested sensor, future research will focus on experimental fabrication, material characterization, controlled surface functionalization, and systematic measurements.

Table 8 compares the suggested SPR sensor’s sensitivity performance with previously published SPR sensing structures. The suggested hybrid SPR configuration is compared to the sensitivity values of current SPR sensors. The suggested sensor achieves a much higher sensitivity of 290.24°/RIU, while the reported structures show sensitivities ranging from 178 to 263.57°/RIU. The bimetallic and TaSe_2_ layers enhance analyte interaction, plasmonic coupling, and electromagnetic field confinement at the sensing interface, which improves sensing performance. The increased sensitivity shows that the suggested structure is superior for extremely precise RI-based biological and biochemical sensing applications.

## 4. Conclusions

For improved biological sensing applications, we theoretically studied a highly sensitive SPR sensor using the TMM. For various biological sensing layers, the effect of Ag layer thickness on sensitivity, DA, and FoM was methodically assessed. The findings show that optimizing plasmon excitation, resonance sharpness, and electromagnetic field distribution at the sensing interface depends critically on Ag thickness. The RI of the SM was affected by Ag thickness. Among the biological layers evaluated, Basal, HeLa, Jurkat, PC12, MM-231, and MCF-7 showed the best Rmin values and the highest sensitivities of 260.49°/RIU, 280.13°/RIU, 290.24°/RIU, and 260.49°/RIU, with an optimized Ag thickness of 42–39 nm, respectively. Additionally, at SM RIs of 1.35 and 1.40, PDs of 192.94 nm and 181.5 nm were obtained. Incorporating the Au layer improves the stability and durability of the Ag plasmonic layer, while the TaSe_2_ layer enhances molecular interaction and field confinement at the sensing interface. The optimized hybrid structure balances improved DA, a low R_min_ value, and high sensitivity. For advanced biosensing applications, such as cancer cell detection, biomolecular analysis, and real-time RI monitoring, this SPR platform offers substantial potential. Its practical applicability in integrated sensing systems can be further validated through future experimental testing.

## Figures and Tables

**Table 1:** RI and thickness used at 633 nm wavelength.

Layers	Thickness	Refractive Index (n + ik)	Ref.
BK7	Semi-infinite	1.5151	[27]
Ag	25–60 nm	0.0803 + 4.2347i	
Au	0–10 nm	0.1378 + 3.6196i	
TaSe_2_	1 nm	3.1591 + 0.51676i	[33]
SM	-	As per Table 2	[35]

**Table 2:** RI of healthy and cancerous cell detection [35].

Cancer Type	Cell Type	Concentration	RI of Cell
Skin cancer	Healthy (Basal)	30–70%	1.360
	Cancerous (Basal)	80%	1.380
Cervical	Healthy (HeLa)	30–70%	1.368
	Cancerous (HeLa)	80%	1.392
Blood	Healthy (Jurkat)	30–70%	1.376
	Cancerous (Jurkat)	80%	1.390
Adrenal Gland	Healthy (PC12)	30–70%	1.381
	Cancerous (PC12)	80%	1.395
Breast I	Healthy (MM 231)	30–70%	1.385
	Cancerous (MM 231)	80%	1.399
Breast II	Healthy (MCF 7)	30–70%	1.387
	Cancerous (MCF 7)	80%	1.401

**Table 3:** Effect of Ag and Au layer thickness optimization on SPR sensor performance, including sensitivity, R_min_, FWHM, DA, and FoM.

Ag Thick. (nm)	Au Thick. (nm)	S (°/RIU)	At n_s_ = 1.35				At n_s_ = 1.40			
			R_min_	FWHM (°)	DA (/°)	FoM (/RIU)	R_min_	FWHM (°)	DA (/°)	FoM (/RIU)
47	0	170.26	2.1 × 10^−5^	2.37	0.421	71.83	1.4 × 10^−2^	3.73	0.268	45.64
46	1	174.52	4.5 × 10^−4^	2.51	0.398	69.53	2.2 × 10^−2^	3.97	0.251	43.96
43	3	181.76	1.8 × 10^−5^	2.92	0.342	62.24	2.5 × 10^−2^	4.64	0.215	39.17
40	5	188.30	3.5 × 10^−4^	3.34	0.299	56.37	2.6 × 10^−2^	5.01	0.199	37.58
37	8	199.54	2.4 × 10^−5^	3.77	0.265	52.93	5.5 × 10^−2^	5.85	0.170	34.11
35	10	206.32	2.6 × 10^−5^	4.03	0.248	51.19	7.8 × 10^−2^	6.13	0.163	33.65

**Table 4:** Optimized Ag thickness and performance parameters of the proposed SPR sensor for different healthy and cancerous cell lines.

RI Analyte	Ag Thick. (nm)	S (°/RIU)	Healthy				Cancerous			
			R_min_	FWHM (°)	DA (/°)	FoM (/RIU)	R_min_	FWHM (°)	DA (/°)	FoM (/RIU)
Basal	34	181.62	2.4 × 10^−4^	4.50	0.221	40.30	1.6 × 10^−3^	2.34	0.427	77.61
HeLa	34	209.12	3.2 × 10^−7^	4.81	0.207	43.47	14 × 10^−2^	5.97	0.167	35.03
Jurkat	33	214.16	4.9 × 10^−4^	5.3	0.188	40.40	2.8 × 10^−4^	6.02	0.166	35.57
PC12	33	234.29	3.9 × 10^−6^	5.54	0.180	42.29	1 × 10^−2^	6.32	0.158	37.07
MM-231	33	254.84	3.5 × 10^−4^	5.74	0.174	44.39	2.4 × 10^−2^	6.51	0.153	39.14
MCF-7	32	262	3.1 × 10^−4^	6	0.166	43.66	18 × 10^−2^	6.77	0.147	38.70

**Table 5:** Maximum sensitivity at optimized Ag thickness with blow 0.30 R_min_ value.

RI Analyte	Ag Thick. (nm)	S (°/RIU)	R_min_ (n_s_ = 1.360–1.387)
Basal	42	260.49	0.156
HeLa	40	280.13	0.114
Jurkat	39	290.24	0.096
PC12	42	260.49	0.156
MM-231	40	280.13	0.114
MCF-7	39	290.24	0.096

**Table 6:** Optimized Ag thickness and performance parameters of the conventional SPR sensor for n_s_ = 1.33 to 1.40.

Conventional Structure	Ag Thick. (nm)	S (°/RIU)	R_min_ at n_s_ = 1.33	R_min_ at n_s_ = 1.40	At n_s_ = 1.33		
					FWHM (°)	DA (/°)	FoM (/RIU)
Ag/SM	52	158.43	2.2 × 10^−4^	8.9 × 10^−3^	1.35	0.740	117.35
Ag/Au/SM	38	185.58	2.3 × 10^−4^	2.1 × 10^−2^	2.64	0.378	70.29
Ag/TaSe_2_/SM	47	170.26	2.1 × 10^−5^	1.4 × 10^−2^	2.33	0.429	73.07

**Table 7:** Impact of performance metrics with ±10% error in thickness of each layer.

Proposed Structure		Proposed Structure Thick.	Possible Errors in a Constituent Layer Thick. (−10%)	Possible Errors in a Constituent Layer Thick. (+10%)
BK7		Semi-infinite	Semi-infinite	Semi-infinite
Ag		35 nm	31.5	38.5
Au		10 nm	9	11
TaSe_2_		1 nm	0.90	1.10
Sensitivity (°/RIU)		206.5	191.68	221.16
R_min_	1.35	2.6 × 10^−5^	3.6 × 10^−2^	1.6 × 10^−4^
	1.40	7.8 × 10^−2^	5.4 × 10^−2^	0.29507
FWHM (°)	1.35	4.03	4.48	6.77
	1.40	6.13	3.5	6.64
DA (/°)	1.35	0.163	0.223	0.147
	1.40	0.248	0.285	0.150
FoM (/RIU)	1.35	51.19	42.78	28.31
	1.40	33.65	63.18	33.30

**Table 8:** Comparison of the performance parameters of the proposed SPR sensor with previously reported SPR sensor structures.

Ref.	Sensor Structure	λ (nm)	RI Range	S (°/RIU)	FWHM (°)	FoM (/RIU)	Fabrication Approach/Cost
[41]	BK7/Ag/ SnSe/SM	633	1.33–1.37	116	NR	NR	Ag thin-film deposition, theoretical TMM, low–moderate cost.
[42]	Prism/Ag/ Graphene	633 nm	1.33–1.41	200	NR	NR	Theoretical design based on FEM; graphene coating; and cost without quantification.
[43]	BK7/ZnO/Ag/BaTiO_3_/WS_2_	633 nm	1.33–1.35	235	3.70	63.51	Theoretical TMM; moderate fabrication complexity; multilayer thin-film deposition.
[44]	BK7/Ag/ Si_3_N_4_/GO	633 nm	1.36–1.401	259	NR	140	Ag, Si_3_N_4_ and GO multilayer deposition; theoretical TMM/FEM; moderate complexity.
[45]	BK7/Ag/ InP/MXene	633 nm	1.33–1.40	263.57	7.45	34.62	PVD/sputtering + InP deposition; theoretical TMM/COMSOL Multiphysics 6.1; and relatively complex.
Proposed work	BK7/Ag/ Au/TaSe_2_	633 nm	1.360–1.401	290.24	6.77	38.70	Ag/Au thin-film deposition with TaSe_2_ coating; theoretical TMM; fabrication cost not experimentally quantified.

## Data Availability

The data set generated is already presented in the manuscript.
